# Targeting the ERK2 D-Recruitment Site with Anticancer Peptides from the DRAMP Database: An Integrated *In Silico* Study

**DOI:** 10.3390/ijms27219532

**Published:** 2026-10-25

**Authors:** Nahlah Makki Almansour, Mahmoud A. A. Ibrahim

**Affiliations:** 1 Department of Biology, College of Science, University of Hafr Al Batin, Hafr Al Batin 1803, Saudi Arabia; 2 Computational Chemistry Laboratory, Chemistry Department, Faculty of Science, Minia University, Minia 61519, Egypt; 3 School of Health Sciences, University of KwaZulu-Natal, Westville Campus, Durban 4000, South Africa; 4 Department of Supportive Requirements, University of Technology and Applied Sciences, Nizwa 611, Oman

**Keywords:** carcinoma disease, ERK2, antimicrobial peptides, molecular dynamics, physicochemical properties

## Abstract

ERK2 is a central regulator of the MAPK pathway, governing cell proliferation, differentiation, and survival, and its aberrant activation is a recognized driver of cancer progression. ATP-competitive ERK2 inhibitors suffer from limited selectivity owing to the highly conserved kinase active site, whereas the ERK2 D-recruitment site (DRS), a non-catalytic docking surface mediating substrate recruitment, offers a more selective route to pathway modulation. Herein, the DRAMP database was screened, yielding 31 peptides with putative anticancer activity within the 5–15 residue range, which were docked towards the ERK2 DRS employing HADDOCK2.4. The protocol was first validated by reproducing the crystallographic binding mode of the co-crystallized KIM-MAP3 peptide. Peptides meeting the applied docking-score threshold were advanced to molecular dynamics simulations, followed by MM-GBSA binding energy calculations. Over 200 ns trajectories, DRAMP03987, DRAMP03983, and DRAMP03985 bound ERK2 more favorably than the reference KIM-MAP3 peptide, with Δ*G*_binding_ values of −71.2, −65.1, and −63.1 kcal/mol, respectively, versus −51.5 kcal/mol. Energetic and structural analyses indicated sustained occupancy of the DRS throughout the simulations, while *in silico* physicochemical profiling revealed preliminary physicochemical characteristics. Collectively, these findings highlight the potential of the identified peptides as ERK2 DRS-binding candidates, warranting further experimental investigation.

## 1. Introduction

The MAPK family consists of highly conserved serine/threonine kinases that serve as important regulators of cellular proliferation, differentiation, survival, programmed cell death, and transformation [1]. ERK2 (Extracellular signal-regulated kinase 2) is a prominent member of the MAPK family and regulates numerous biological processes in eukaryotic cells [2,3,4,5]. Aberrant activation of the MAPK signaling cascade, particularly the Ras/Raf/MEK/ERK pathway, is strongly implicated in the initiation and progression of cancer [6]. Activation of ERK2 proceeds through successive phosphorylation steps in the MAPK cascade, after which the kinase enters the nucleus and phosphorylates transcription factors, thereby inducing effector proteins that govern proliferation and survival [7]. Consequently, sustained or abnormal ERK2 activation participates directly in oncogenesis [7]. Dysregulation of ERK2 signaling has been associated with the development and progression of several malignancies and proliferative disorders, with hepatocellular carcinoma (HCC) being a prominent example [8,9,10]. Although several classes of ERK2 inhibitors, including allosteric, ATP-competitive, and covalent inhibitors, have been developed, no FDA-approved therapy specifically targeting ERK2 is currently available [11,12]. ERK2 is characterized by two regulatory docking sites, the DRS (D-recruitment site) and the FRS (F-recruitment site), which facilitate interactions with upstream activators and downstream substrates. However, the selective pharmacological targeting of these protein–protein interaction interfaces remains challenging [13,14,15]. Recent studies have demonstrated that inhibition of the ERK2 DRS represents a promising strategy to enhance inhibitor selectivity and mitigate the drawbacks associated with ATP-binding site inhibition [16,17]. Selective modulation of ERK2 may instead be achieved using short peptides that target protein-protein interfaces, thereby avoiding the conserved ATP pocket that limits the specificity of conventional inhibitors [18,19]. Peptides based on D-site motifs (kinase interaction motifs, KIMs) bind the ERK2 DRS, occluding substrate engagement and interrupting downstream signalling [13]. Crystallographic and biochemical analyses have shown that D-site peptides dock within a groove comprising a hydrophobic patch and an acidic common docking region, an interaction underpinned by salt bridges and H-bonds with key DRS residues [20]. Collectively, these findings demonstrate that ERK2 signalling can be selectively modulated through its docking surface, establishing the DRS as a druggable allosteric site.

Peptide therapeutics have emerged as a promising class of anticancer agents, combining high target selectivity with tractability to structure-guided optimization [21,22,23,24]. Furthermore, the rapid metabolism and clearance of peptide-based therapeutics often limit their accumulation in specific organs, thereby lessening the risk of systemic toxicity [25,26,27]. Despite their potential advantages in recognizing protein–protein interaction interfaces, peptide therapeutics face several challenges, including susceptibility to proteolytic degradation, limited oral bioavailability, rapid systemic clearance, and restricted intracellular accessibility, which may compromise their therapeutic efficacy. These limitations are particularly relevant to intracellular proteins such as ERK2, for which efficient cellular delivery is essential. Nevertheless, the identification of promising peptide candidates may provide valuable molecular scaffolds for subsequent optimization to overcome these limitations and improve their therapeutic potential.

Antimicrobial peptides (AMPs) represent the most thoroughly characterized class of bioactive peptides and provide an abundant source of molecules spanning a wide range of biological activities [28]. AMPs are often associated with favorable safety profiles; however, their toxicity and off-target effects may vary depending on peptide sequence and context, and require further evaluation [29]. In this context, the data repository of antimicrobial peptides (DRAMP) is one of the most comprehensive resources of AMPs [30,31]. DRAMP comprises peptides derived from diverse biological sources, including animals, microorganisms, plants, and synthetic sources, and exhibits a wide range of biological activities [30]. Of note, the putative role of AMPs in targeting the ERK2 DRS has not yet been systematically examined, positioning the current study within a largely uncharted research space.

**Figure 1:**
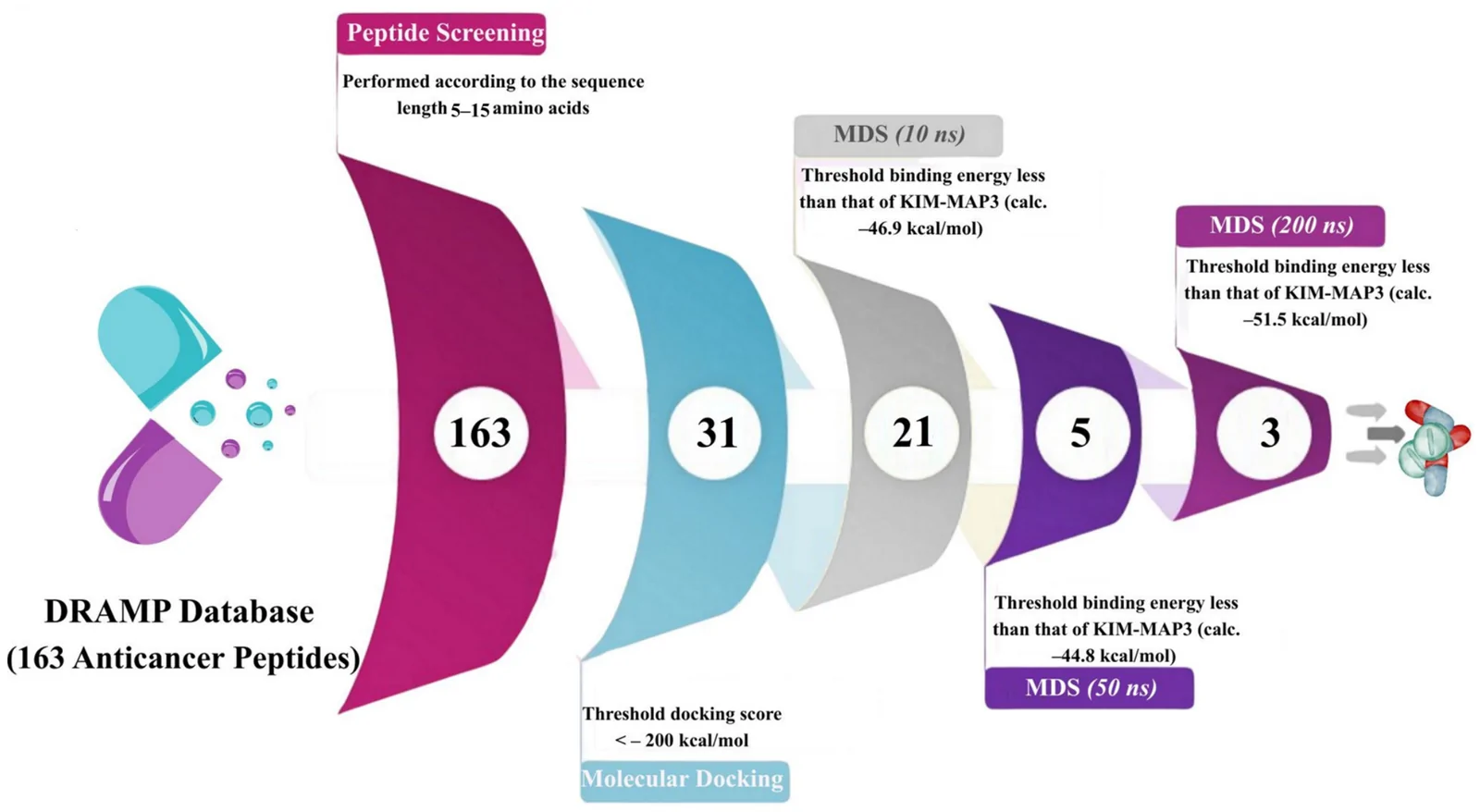
Flowchart diagram of the applied computational methods to identify the promising DRAMP peptides against the DRS of ERK2.

In this study, 163 peptides annotated with anticancer activity were retrieved from the DRAMP database, which contains 22,259 entries. Following sequence-based filtering and the application of a peptide length criterion of 5–15 amino acid residues, 31 candidates were selected for computational evaluation of their potential binding interactions with the ERK2 DRS. The 3D structures of the selected peptides were generated using AlphaFold2 and docked into the DRS of ERK2 using HADDOCK (version 2.4) software. The top-ranked peptides bound to ERK2 subsequently underwent MD simulations (MDS) throughout 200 ns, complemented by MM-GBSA calculations. The most favorable DRAMP peptides were subsequently subjected to post-simulation analyses and physicochemical characterization. Figure 1 illustrates the computational workflow used to screen the DRAMP database for ERK2 DRS, as described stepwise in the Computational Methodology section. Taken together, the present findings support the identified peptides as computationally selected ligands of the ERK2 DRS that merit further experimental investigation.

## 2. Results and Discussion

### 2.1. DRAMP Database Filtration

In the current study, a total of 22,259 curated AMPs were obtained from the DRAMP database. Entries were first restricted to those carrying an anticancer activity annotation, which produced a preliminary set of 163 peptides. Peptides lacking complete sequence information or containing non-standard AA were excluded from further analysis. Applying a length criterion of 5–15 amino acids narrowed the selection to the 31 peptides (Table S1). This length range was selected to prioritize peptides with favorable developability and synthetic feasibility while maximizing the likelihood of productive interactions with the ERK2 DRS. Peptides shorter than five residues were excluded because they generally provide insufficient interaction interfaces for stable binding, whereas longer peptides were excluded to improve synthetic tractability and pharmaceutical developability. The 3D conformations of the selected peptides were generated utilizing the AlphaFold2 platform. The top-ranked model, selected by predicted confidence score, was taken forward as the representative structure of each peptide. It is worth mentioning that the anticancer annotation of DRAMP peptides was used exclusively as an initial screening criterion and did not imply a mechanistic association with ERK2 signaling. Given that anticancer peptides may exert their biological effects through diverse mechanisms, the present computational study aimed to evaluate their structural compatibility with the predefined ERK2 DRS without assuming that their reported anticancer activities are mediated by ERK2 modulation.

### 2.2. Docking Validation

**Figure 2:**
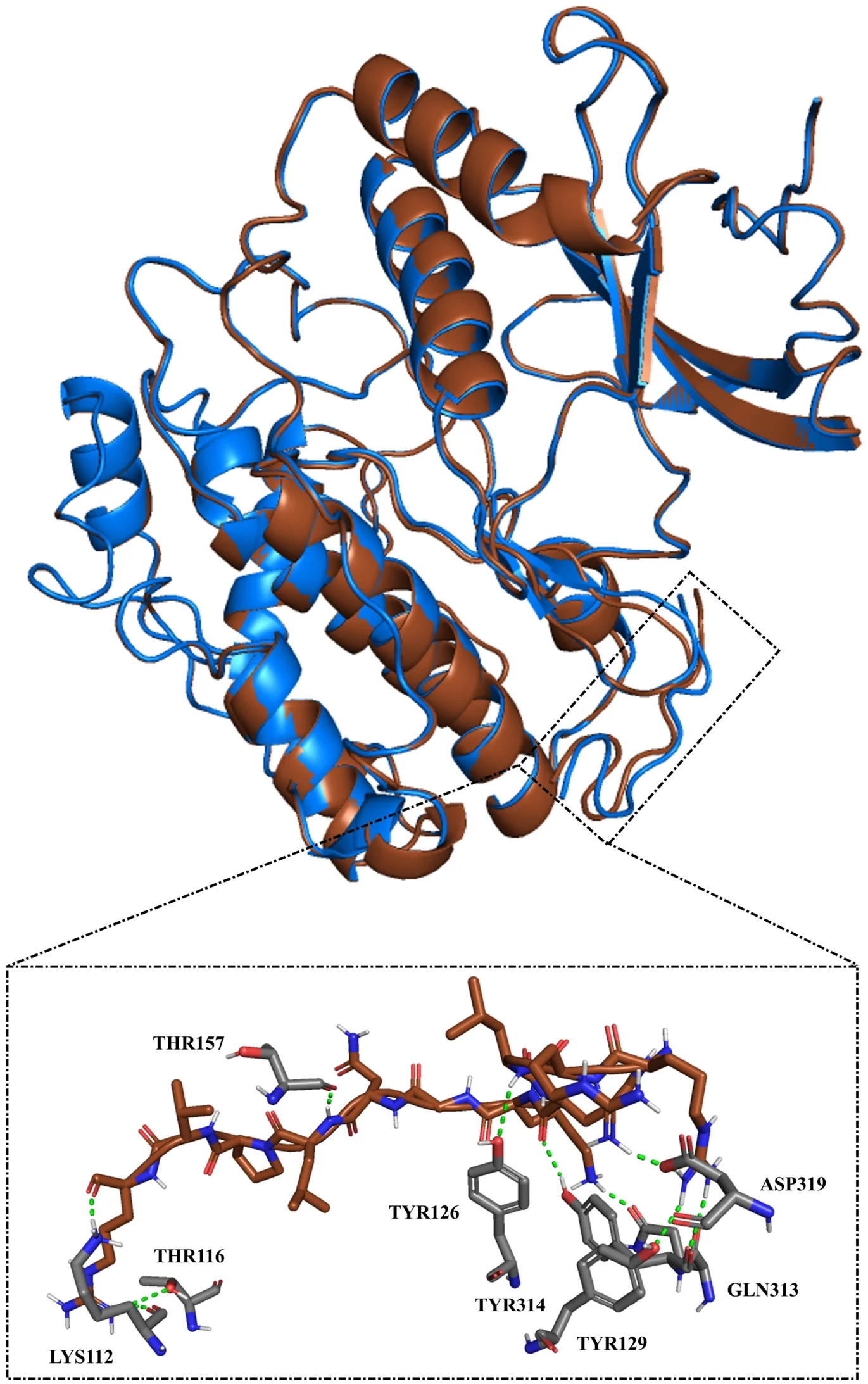
Overlay of the docked (blue) and experimentally determined (brown) conformations of the KIM-MAP3 peptide bound to the ERK2 DRS.

A docking validation analysis was undertaken to assess whether HADDOCK2.4 could reliably predict peptide interaction geometries at the ERK2 DRS. For validation purposes, the co-crystallized KIM-MAP3 peptide was prepared and re-docked towards the ERK2 DRS (Figure 2). As disclosed in Figure 2, the docked pose superimposed well on the experimental structure (RMSD = 1.23 Å), confirming that the protocol recovers the native binding mode. The KIM-MAP3 peptide additionally returned a HADDOCK score of −322.1 against the ERK2 DRS. Analysis of the predicted docking conformation showed that key ERK2 DRS residues (GLN313, LYS112, TYR126, THR116, ASP319, THR157, TYR314, and TYR129) established ten H-bonds with the KIM-MAP3 peptide (ARG74, LEU71, ARG65, LYS68, ARG64, and GLN67), with bond lengths between 1.5 and 2.7 Å.

### 2.3. Binding Recognition of Selected Peptides at the ERK2 DRS

**Figure 3:**
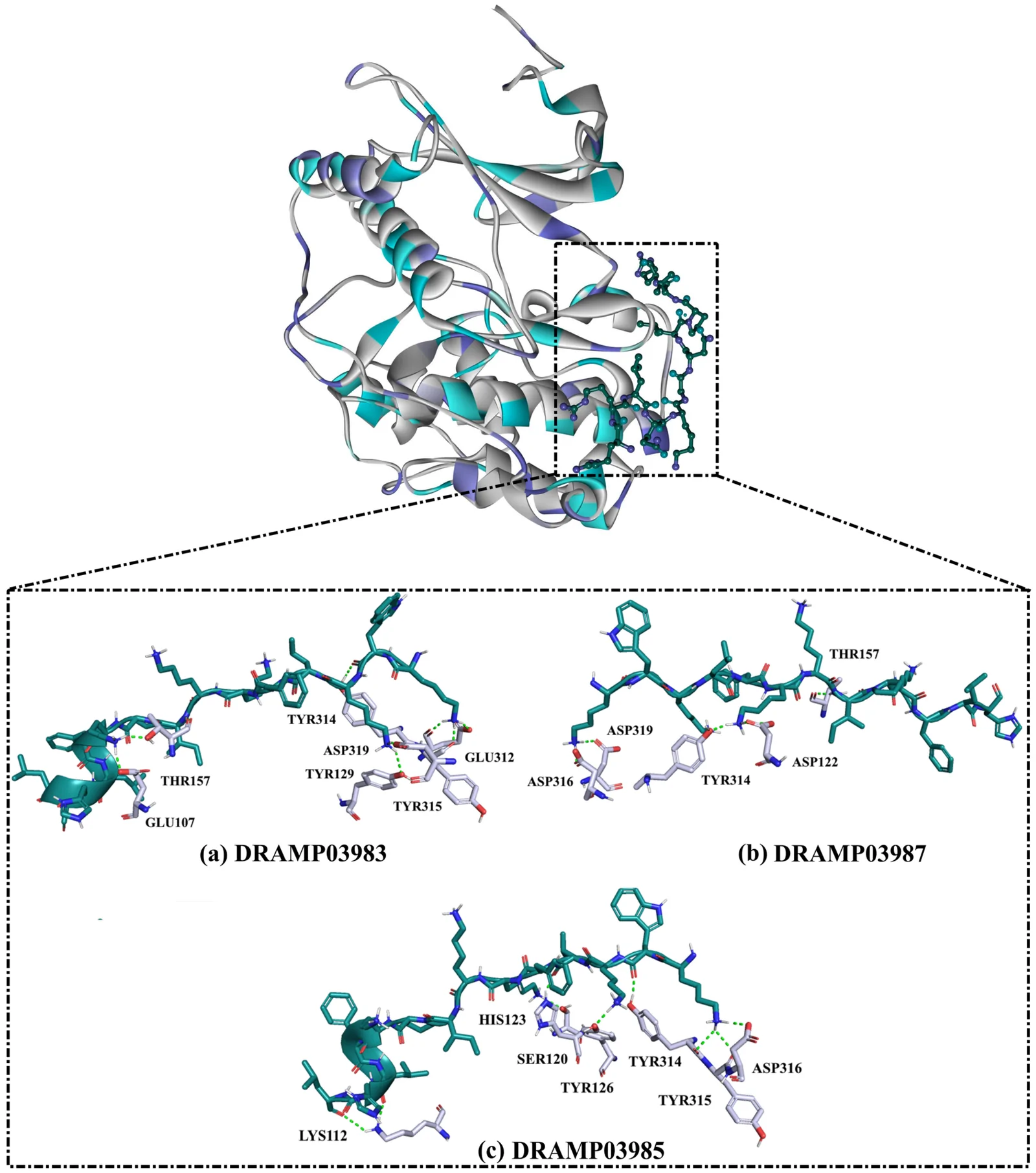
Three-dimensional interaction diagrams illustrating the binding of (**a**) DRAMP03983, (**b**) DRAMP03987, and (**c**) DRAMP03985 within the ERK2 DRS.

A curated library comprising 31 DRAMP peptides with anticancer potential was docked into the ERK2 DRS using the validated HADDOCK2.4 docking protocol. The calculated HADDOCK scores ranged from −85.6 to −318.6, with 21 of the 31 screened peptides exhibiting scores below −200. This empirically selected threshold was employed as a practical prioritization criterion to retain approximately two-thirds of the peptide candidates for subsequent MDS and MM-GBSA calculations, rather than as a validated cutoff for identifying ERK2 DRS binders. As HADDOCK scores are derived from a weighted empirical scoring function and do not represent physical binding free energies, they were interpreted solely as relative ranking metrics to guide the selection of candidates for further computational evaluation. Figure S1 depicts the 3D contacts formed between the investigated peptides and critical ERK2 residues. Most peptides adopted a comparable binding orientation, characterized by a conserved H-bond with THR157 and additional H-bonding interactions across the DRS (Figure S1). The predicted docking scores and intermolecular H-bond interactions of the three most promising peptides against ERK2 DRS are compiled in Table 1. Figure 3 represents the predicted binding poses of the three selected peptides in complex with the ERK2 DRS. Of note, these three peptides were chosen based on their MM-GBSA binding energies computed over 200 ns MDS, as elaborated in the forthcoming sections.

DRAMP03983 exhibits bioactivity that supports its potential anticancer relevance, particularly in the context of peptide-mediated interference with cancer-associated protein–protein interaction [32]. The docking results revealed a pronounced interaction of DRAMP03983 with the ERK2 DRS, corresponding to a docking score of −318.6 kcal/mol. Analysis of the docking pose indicated that DRAMP03983 engaged the ERK2 DRS through ten H-bonds involving GLU107, TYR129, THR157, GLU312, TYR314, TYR315, and ASP319, with corresponding distances of 1.5–2.2 Å (Figure 3).

DRAMP03987 is a bioactive AMP with emerging relevance in anticancer research [32]. DRAMP03987 ranked second among the screened peptides, yielding a docking score of −292.3 kcal/mol for binding to the ERK2 DRS. As shown in Figure 3, the strong docking performance of DRAMP03987 was rationalized by the formation of five intermolecular H-bonds with ASP122, TYR314, THR157, ASP316, and ASP319 within the ERK2 DRS, displaying bond lengths of 1.5–2.2 Å.

DRAMP03985 is a DRAMP-annotated AMP with emerging relevance in anticancer research [32]. DRAMP03985 ranked favorably in the docking analysis, with a docking score of −273.9 kcal/mol and an extensive H-bonding network comprising nine interactions with LYS112, SER120, TYR126, TYR314, TYR315, and ASP316 (Figure 3). Overall, the docking results supported plausible binding of the identified DRAMP peptides to the ERK2 DRS, based on both their scoring profiles and interaction patterns.

Overall, comparative analysis of the docking results showed that DRAMP03983 achieved a docking score of −318.6 kcal/mol, closely matching that of the reference peptide KIM-MAP3 (calc. −322.1 kcal/mol). DRAMP03987 and DRAMP03985 yielded less favorable, yet still comparatively strong, docking scores of −292.3 and −273.9 kcal/mol, respectively.

### 2.4. MD Simulations

**Figure 4:**
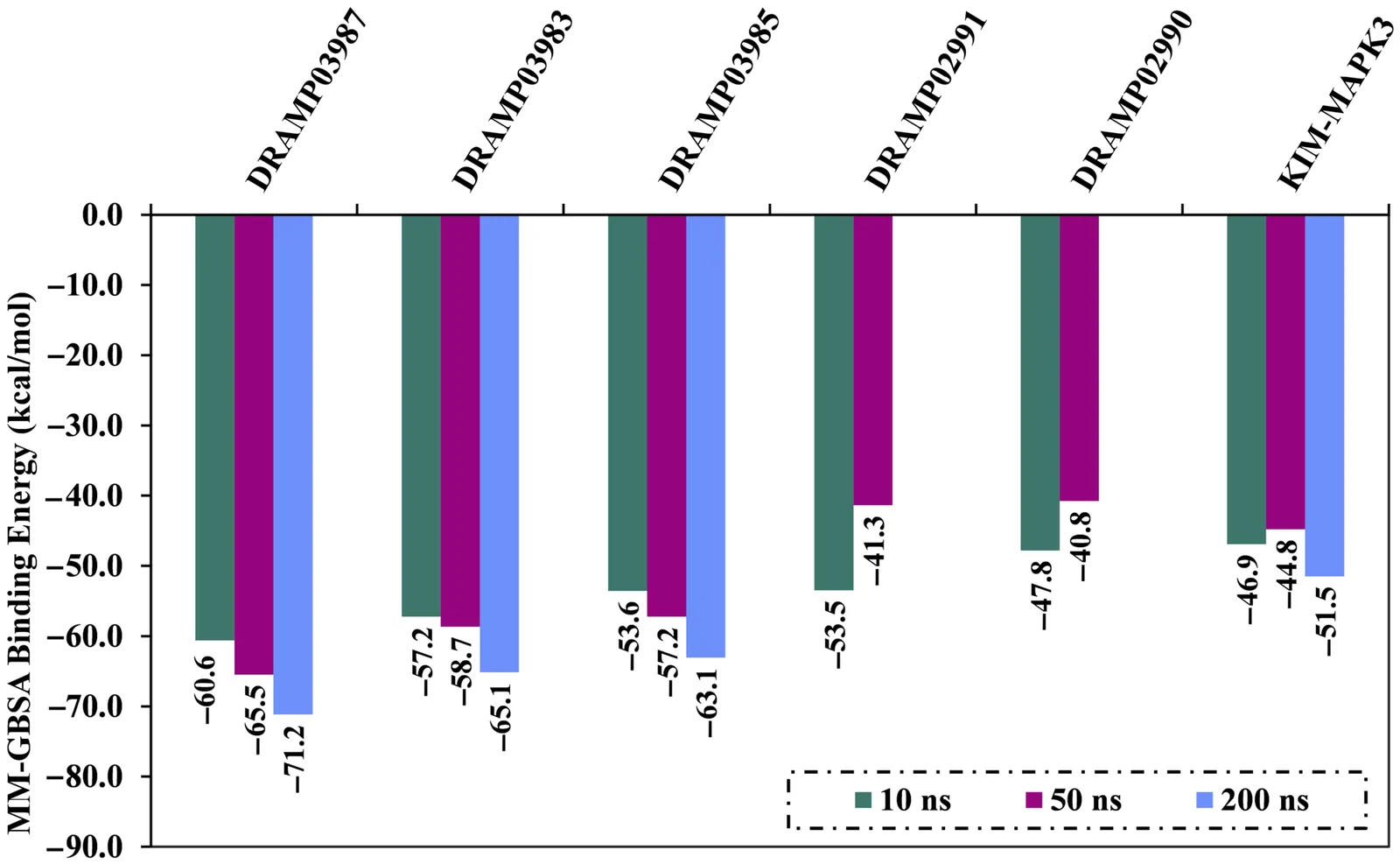
Estimated binding energies of the most promising peptides-ERK2 complexes at 10, 50, and 200 ns simulation intervals.

MDS has emerged as a cornerstone of contemporary pharmacological and structural biology research [33,34]. Molecular dynamics simulations provide a time-resolved description of peptide–protein complexes, thereby illuminating interaction patterns, conformational behavior, and complex stability. As a result, the 21 DRAMP peptides exhibiting docking scores < −200.0 kcal/mol bound to the ERK2 DRS further underwent 10 ns MDS, and their MM-GBSA binding energies were computed (Table S3). A preliminary 10 ns simulation was performed for each complex as a rapid screen for structural stability. As summarized in Table S3, five peptides showed binding affinities exceeding that of KIM-MAP3 (Δ*G*_binding_ = −46.9 kcal/mol), with Δ*G*_binding_ values between −47.8 and −57.2 kcal/mol. To enhance the reliability of the evaluated binding affinities, the identified peptide-ERK2 complexes underwent a prolonged 50 ns MDS, along with Δ*G*_binding_ estimations (Figure 4). Among the evaluated peptides, DRAMP03987, DRAMP03983, and DRAMP03985 towards ERK2 DRS achieved greater binding affinities than KIM-MAP3 (Δ*G*_binding_ = −44.8 ± 9.3 kcal/mol) (Figure 4). Numerically, the estimated Δ*G*_binding_ values over 50 ns MDS for DRAMP03987-, DRAMP03983-, and DRAMP03985-ERK2 complexes were −65.5 ± 9.6, −58.7 ± 8.0, and −57.2 ± 6.5 kcal/mol, respectively. To assess the reproducibility and statistical robustness of the computational findings, two independent 50 ns MDS were conducted for DRAMP03987, DRAMP03983, and DRAMP03985 complexed with the ERK2 DRS, followed by MM-GBSA binding energy calculations (Table S4). The estimated Δ*G*_binding_ values exhibited limited variation across the independent trajectories, suggesting consistent energetic behavior. For instance, DRAMP03987 yielded Δ*G*_binding_ values of −65.5 ± 9.6 and −61.5 ± 7.5 kcal/mol across the two independent simulations. Based on these consistent energetic trends, the MDS were subsequently extended to 200 ns for the investigated peptide-ERK2 complexes, followed by MM-GBSA binding energy evaluations (Figure 4). Of note, the calculated binding energies of the identified peptide-ERK2 complexes remained consistent over the 50 and 200 ns MDS. Compared with KIM-MAP3 (Δ*G*_binding_ = −51.5 ± 9.9 kcal/mol), DRAMP03987, DRAMP03983, and DRAMP03985 exhibited more negative MM-GBSA binding energy estimates toward the ERK2 DRS over the 200 ns MDS, with average Δ*G*_binding_ values of −71.2 ± 11.9, −65.1 ± 8.6, and −63.1 ± 11.4 kcal/mol, respectively. Notably, DRAMP03983, DRAMP03985, and DRAMP03987 are closely related C-terminal truncation variants associated with the P18 peptide family, rather than independent peptide leads [32]. Their calculated MM-GBSA binding energy estimates became progressively more negative with decreasing peptide length, suggesting a possible influence of C-terminal truncation on the modeled peptide-ERK2 interactions. Further investigations involving full-length P18 would provide a more comprehensive assessment of peptide-length effects. Moreover, the exclusion of entropic contributions from the MM-GBSA calculations warrants cautious interpretation of the observed energetic trends, which could be further evaluated through experimental binding affinity measurements. Collectively, these outcomes highlighted DRAMP03987, DRAMP03983, and DRAMP03985 as potential ERK2 DRS-targeting peptides and candidates for further experimental validation. Although the 200 ns MDS and MM-GBSA calculations demonstrated sustained interactions and favorable estimated binding energies of the selected peptides within the ERK2 DRS, these findings did not independently establish binding-site specificity, as the simulations were initiated from DRS-directed docking poses. Nevertheless, the results provided valuable insights into the relative stability and binding energetics of the investigated peptide-ERK2 complexes under the modeled binding configurations.

**Figure 5:**
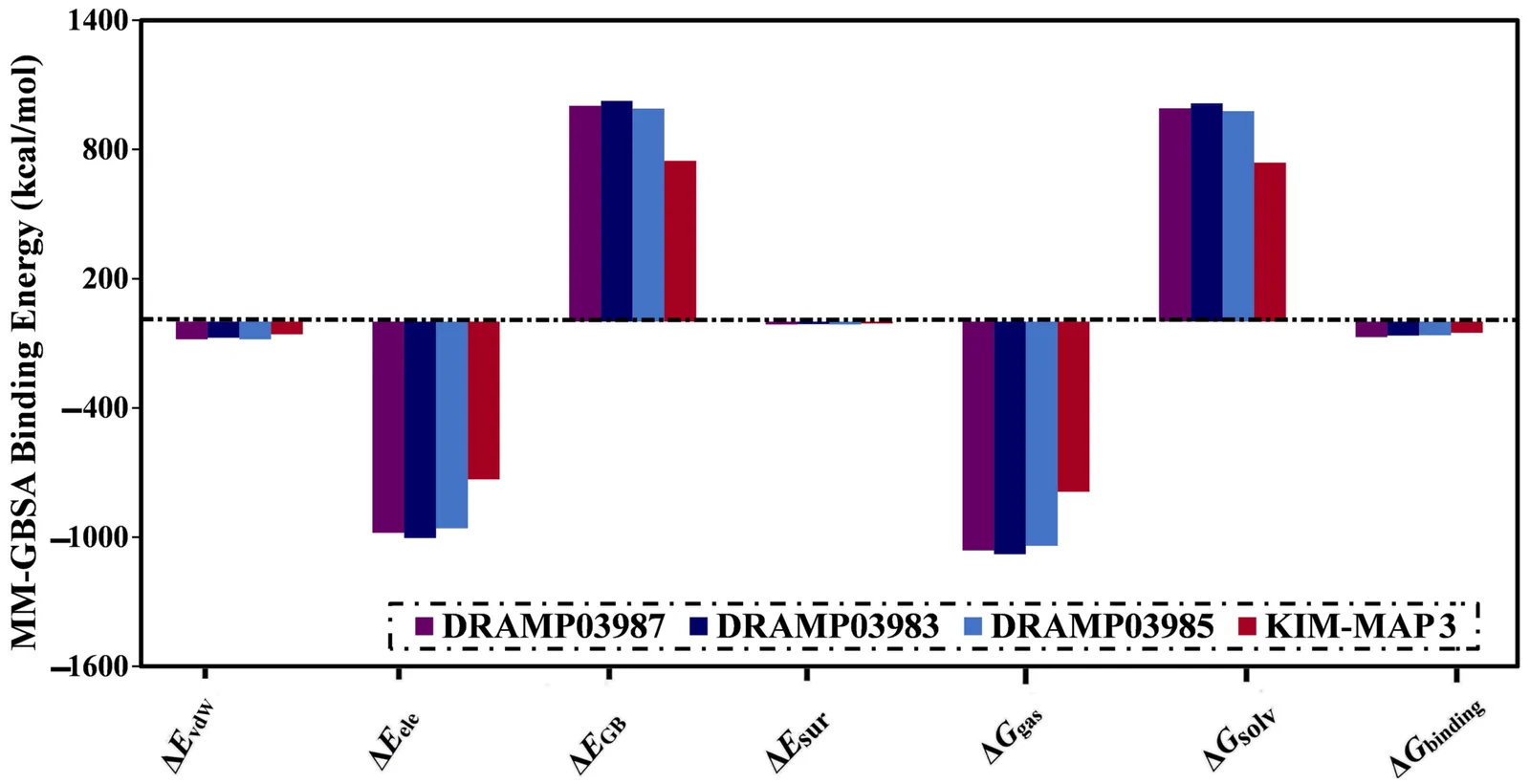
Δ*G*_binding_ decomposition of DRAMP03987, DRAMP03983, DRAMP03985, and KIM-MAP3 complexed with the ERK2 DRS across 200 ns MD trajectories.

To elucidate the energetic basis of peptide recognition at the ERK2 DRS, Δ*G*_binding_ was decomposed to identify the dominant interaction forces. Figure 5 depicts the dominant interaction components governing the binding of the investigated peptides at the ERK2 DRS over 200 ns MDS. As depicted in Figure 5, *E*_ele_ was the predominant contributor to the binding affinities of DRAMP03987, DRAMP03983, DRAMP03985, and KIM-MAP3 complexed with ERK2 DRS, yielding values of −979.8, −1004.4, −959.4, and −731.7 kcal/mol, respectively. Additionally, *E*_vdW_ contributed favorably to the Δ*G*_binding_, with values of −81.8, −74.6, −81.5, and −58.1 kcal/mol for DRAMP03987, DRAMP03983, DRAMP03985, and KIM-MAP3 bound to the ERK2 DRS, respectively.

The MM-GBSA energy decomposition revealed substantial *E*_ele_ contributions, alongside favorable *E*_vdW_ interactions, to the estimated binding energetics of the modeled peptide-ERK2 complexes. However, the highly cationic nature of the investigated peptides may also promote nonspecific interactions with negatively charged protein surfaces. Moreover, MM-GBSA estimates are sensitive to the treatment of electrostatic and solvation effects, while the exclusion of configurational entropy limits their quantitative interpretation. Therefore, the calculated binding energies should be considered approximate energetic estimates rather than definitive evidence of high binding affinity or inhibitory potency. Given the structural similarities between the ERK2 DRS and the corresponding docking regions of related MAPKs, particularly ERK1, the present findings did not establish selective recognition of ERK2. Further comparative investigations involving ERK1, other MAPKs, and unrelated protein interfaces would provide valuable insights into the binding preferences and molecular selectivity of the identified peptides.

**Figure 6:**
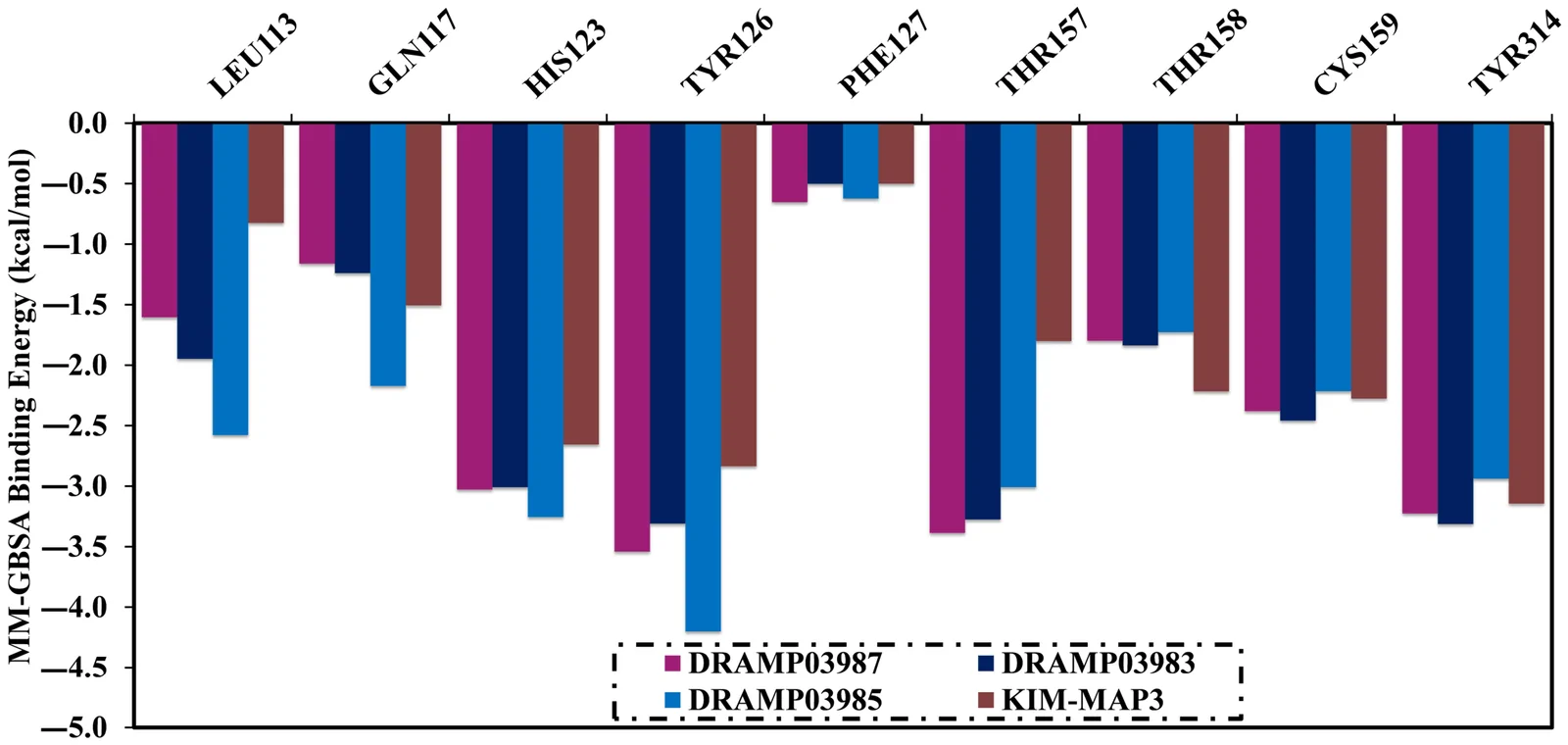
Per-residue contributions to Δ*G*_binding_ of DRAMP03987-, DRAMP03983-, DRAMP03985-, and KIM-MAP3-ERK2 complexes over 200 ns simulation trajectories.

Moreover, per-residue energy decomposition analysis was conducted for DRAMP03987-, DRAMP03983-, DRAMP03985-, and KIM-MAP3-ERK2 complexes over a 200 ns MDS to identify proximal residues that play critical roles in peptide recognition. Figure 6 represents the energy contributions of residues exhibiting binding energy values lower than −0.5 kcal/mol. A −0.5 kcal/mol cutoff was used to highlight notable residues; however, this approximate threshold may influence selection and is interpreted comparatively. Notably, HIS123, TYR314, TYR126, and THR157 participated considerably in the interactions of DRAMP03987, DRAMP03983, DRAMP03985, and KIM-MAP3 with DRS of ERK2. For example, TYR126 exposed substantial stabilizing contributions, with Δ*G*_binding_ values of −3.5, −3.3, −4.2, and −2.8 kcal/mol for DRAMP03987, DRAMP03983, DRAMP03985, and KIM-MAP3, respectively. The residue THR157 consistently contributed favorably to peptide binding at the ERK2 DRS, with Δ*G*_binding_ contributions of −3.4 kcal/mol for DRAMP03987, −3.3 kcal/mol for DRAMP03983, −3.0 kcal/mol for DRAMP03985, and −1.8 kcal/mol for KIM-MAP3.

Most clinically advanced ERK inhibitors, such as SCH772984 and ulixertinib (BVD-523), act by targeting the kinase ATP-binding pocket, achieving very high potency (low nanomolar to sub-nanomolar) [35,36]. However, because this pocket is highly conserved among kinases, achieving selectivity remains challenging, and resistance associated with ATP-site targeting has emerged as a recognized clinical concern. In contrast, the peptide candidates identified in this study were predicted to bind the DRS, a distinct surface on ERK2 that mediates substrate recognition rather than catalytic activity. This site has previously been explored as a therapeutic target; for example, small molecules such as BI-78D3 and cyclic guanidino compounds inhibit ERK2 by interacting with the DRS, including residue ASP319 [13]. The observation that these peptide candidates were also predicted to interact with ASP319 supports the plausibility of the proposed binding mode. Although binding energies cannot be directly compared across computational and experimental studies, the recurring involvement of key DRS residues, including ASP319, TYR314, and THR157, in both previously reported inhibitors and the predicted peptide-ERK2 complexes described here further substantiates the DRS as a viable non-ATP-competitive target and places these peptide candidates within an established ERK2-targeting strategy.

### 2.5. Post-MD Analyses

Post-simulation analyses were executed throughout the entire 200 ns MDS to assess the stability of DRAMP03987, DRAMP03983, DRAMP03985, and KIM-MAP3 within the ERK2 DRS, and their performance was compared with the reference KIM-MAP3 peptide. The post-MD estimation metrics included binding energy per trajectory, H-bond analysis, radius of gyration (Rg), and root-mean-square fluctuation and deviation (RMSF and RMSD, respectively).

#### 2.5.1. Trajectory-Based Binding Energy Analysis

**Figure 7:**
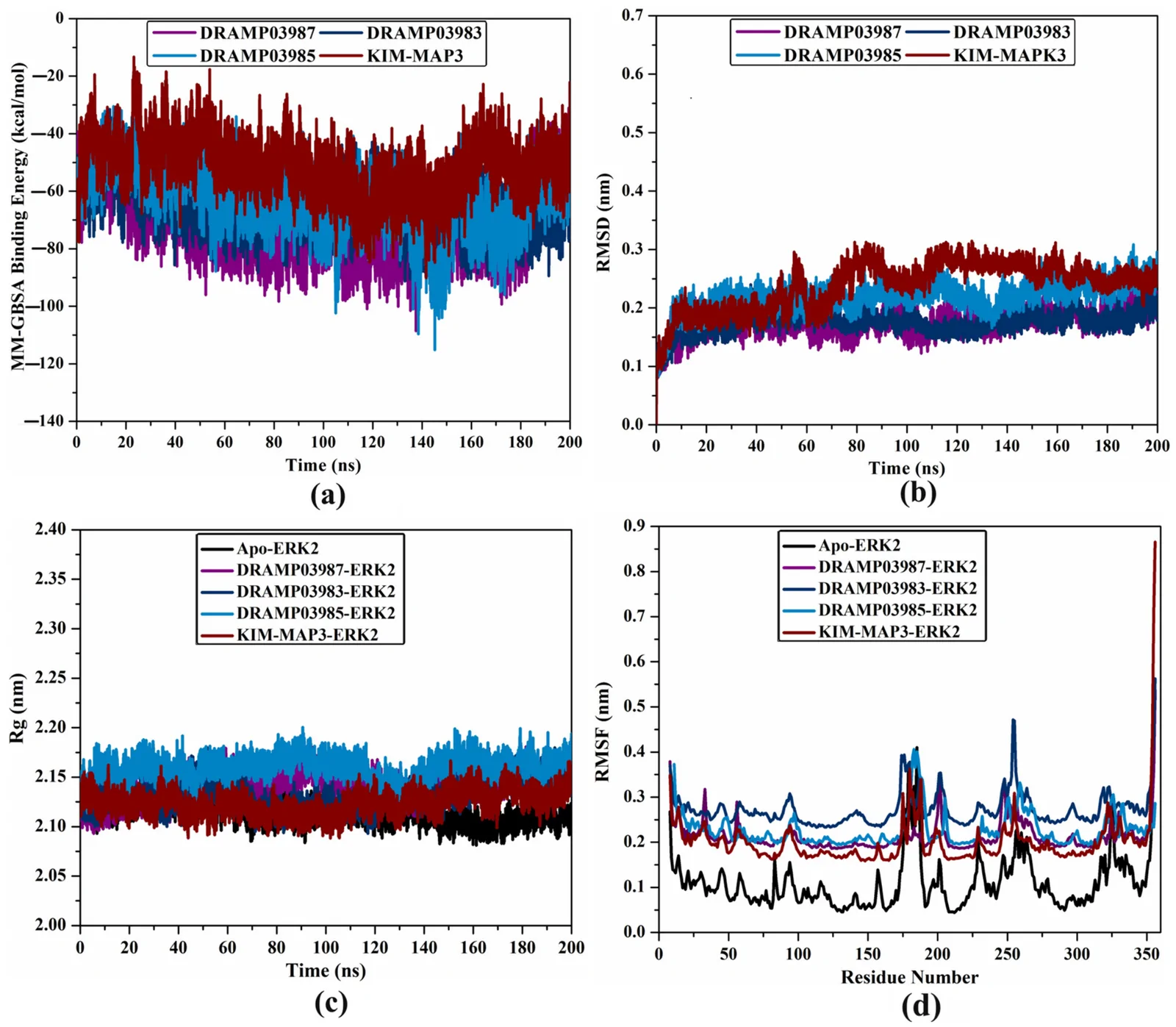
(**a**) Binding energy per trajectory, (**b**) RMSD, (**c**) Rg, and (**d**) RMSF analyses for apo-ERK2 (black) and DRAMP03987 (purple), DRAMP03983 (navy), DRAMP03985 (light blue), and KIM-MAP3 (dark red) bound to ERK2 DRS throughout a 200 ns MDS.

The binding energy profiles of the identified DRAMP peptide-ERK2 complexes and the KIM-MAP3 reference complex were examined throughout the 200 ns MD trajectories to assess their energetic stability (Figure 7a). As disclosed in Figure 7a, DRAMP03987, DRAMP03983, and DRAMP03985 in complex with the ERK2 DRS exhibited more negative binding energies than KIM-MAP3 with Δ*G*_binding_ values of −71.2, −65.1, −63.1, and −51.5 kcal/mol, respectively. The binding energy profiles of the identified peptides remained relatively stable over the 200 ns simulations, with only modest fluctuations observed for the peptide-ERK2 complexes.

#### 2.5.2. RMSD Analysis

RMSD provides a quantitative measure of structural stability and time-dependent conformational changes in protein-ligand complexes during MDS [37]. The structural stability of the identified DRAMP peptide-ERK2 complexes and the KIM-MAP3 reference complex was further assessed using RMSD analysis over the 200 ns MD trajectories (Figure 7b). From Figure 7b, the average RMSD values were 0.18, 0.18, 0.22, and 0.24 nm for the DRAMP03987, DRAMP03983, DRAMP03985, and KIM-MAP3 bound to ERK2 DRS, respectively. The consistently low RMSD values implied that the investigated complexes maintained structural integrity relative to their initial conformations.

#### 2.5.3. Rg Analysis

Rg analysis was conducted to monitor the temporal evolution of the structural compactness and stability of apo-ERK2 and the peptide-ERK2 complexes over 200 ns MDS (Figure 7c). As illustrated in Figure 7c, apo-ERK2 and DRAMP03987, DRAMP03983, DRAMP03985, and KIM-MAP3 bound to ERK2 DRS exhibited comparable Rg values of approximately 2.1 nm. These results indicated that binding of the identified peptides preserved the overall structural compactness of the ERK2 DRS and did not induce significant conformational changes within the DRS.

#### 2.5.4. RMSF Analysis

RMSF is a significant parameter employed to measure the flexibility and dynamic fluctuations of protein residues during MDS [38]. Therefore, RMSF analysis was conducted for the apo-ERK2 and the investigated peptides in complex with the ERK2 DRS over a 200 ns MDS (Figure 7d). According to Figure 7d, the average RMSF value for apo-ERK2 was approximately 0.4 nm, relatively lower than that of the peptide-bound complexes, reflecting the increased local flexibility associated with peptide binding at the DRS interface. Nonetheless, all peptide-bound complexes exhibited relatively low residual fluctuations, confirming their structural stability upon binding to ERK2 DRS.

#### 2.5.5. Analysis of H-Bond

**Figure 8:**
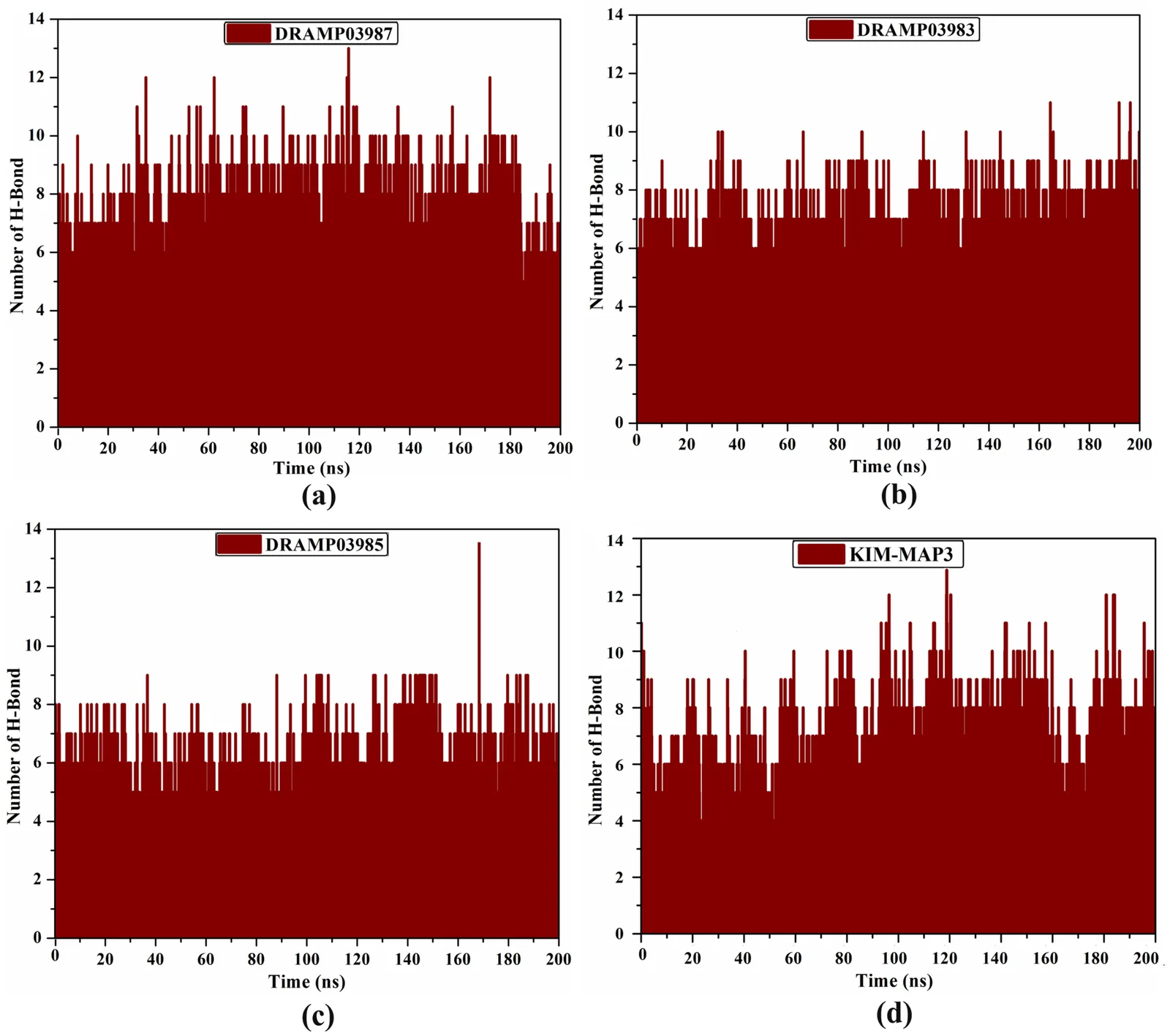
Number of H-bonds of (**a**) DRAMP03987-, (**b**) DRAMP03983-, (**c**) DRAMP03985-, and (**d**) KIM-MAP3-ERK2 complexes throughout the 200 ns MDS.

The H-bond numbers established within inhibitor-receptor complexes contribute to interaction stability but are not solely determinative [39]. The H-bonding profiles of the identified DRAMP peptide-ERK2 complexes were evaluated throughout the 200 ns simulation period to assess their interaction patterns (Figure 8). Figure 8 demonstrates that DRAMP03987, DRAMP03983, DRAMP03985, and KIM-MAP3 established average H-bond counts of eight, seven, six, and seven with the ERK2 DRS, respectively. The maintenance of these interactions throughout the 200 ns MD simulations suggested stable and persistent peptide-protein contacts.

### 2.6. Physicochemical Characteristics

To comprehensively evaluate the advantages of the identified DRAMP peptides, their physicochemical profiles were systematically examined using the Expasy ProtParam tool (Table 2). DRAMP03987, DRAMP03983, DRAMP03985, and KIM-MAP3 disclosed MW of 1713.19, 1897.43, 1826.35, and 1336.61 Da, respectively. The predicted theoretical isoelectric points of DRAMP03987, DRAMP03983, and DRAMP03985 were >7.0, indicating that these peptides had alkaline character and basic physicochemical properties. Peptide stability under physiological conditions was checked for the identified DRAMP peptides using the instability index. DRAMP03987, DRAMP03983, and DRAMP03985 showed instability index values of −17.61, −13.93, and −15.64, respectively, suggesting favorable sequence-derived stability indices under *in vitro* approximation [40]. In contrast, KIM-MAP3 displayed a substantially elevated instability index of 110.38, suggesting a pronounced tendency toward structural instability. The aliphatic index of the inspected peptides spanned from 90.0 to 111.43, reflecting their favorable thermostability. Ultimately, the GRAVY values for DRAMP03987, DRAMP03983, DRAMP03985, and KIM-MAP3 were −0.577, −0.127, −0.264, and −1.327, respectively, consistent with their hydrophilic character. It is worth mentioning that the ProtParam-derived descriptors provide preliminary insights into the sequence-dependent physicochemical properties of the identified peptides rather than definitive evidence of drug-likeness or pharmaceutical developability. These parameters do not establish proteolytic stability, membrane permeability, intracellular accessibility, or pharmacokinetic behavior, which require further experimental assessment. Moreover, the cationic nature and hydrophobic amino acid residues of the identified peptides may promote interactions with cellular membranes, potentially contributing to membrane-disruptive effects independent of ERK2-mediated activity. However, their ability to cross cellular membranes and reach intracellular ERK2 remains unverified. Therefore, further experimental investigations assessing membrane integrity, cellular uptake, intracellular localization, and ERK2 pathway modulation would provide valuable insights into their biological mechanisms and therapeutic potential.

## 3. Materials and Methods

### 3.1. Preparation of DRAMP

The DRAMP database contains 22,259 curated AMPs with experimentally validated sequences and key annotations, serving as a reliable resource for peptide-based drug discovery and computational screening [30]. An initial set of 163 peptides annotated with anticancer activity was retrieved from the DRAMP database. The anticancer annotations were obtained from the DRAMP database and used as an initial selection criterion. However, these annotations were not considered evidence of ERK2-specific activity or a common anticancer mechanism among the selected peptides. Only short peptides comprising 5–15 amino acids with reported anticancer potential, yielding 31 peptides, were selected and prepared for docking calculations. Peptides within the 5–15 residue range were prioritized, as this length reconciles acceptable pharmacokinetic properties with retention of the structural features required for target engagement [41]. The 3D structures of the shortlisted peptides were anticipated by AlphaFold2 prior to docking [42]. While AlphaFold2 can provide reasonable initial peptide conformations, it may not fully capture the conformational diversity of short, flexible peptides [42,43]. Ionization states of the modelled DRAMP peptides were determined utilizing PROPKA [44].

### 3.2. ERK2 Preparation

Coordinates for the ERK2-KIM(MAP3) complex (PDB ID: 2FYS, 2.50 Å [45]) were employed as the structural basis for all docking calculations and as the template for all computational investigations. KIM-MAP3 was selected as the reference peptide because it was the only experimentally determined, co-crystallized peptide bound to the ERK2 docking recruitment site (DRS) available at the time of this study. This structurally validated complex served as a reliable benchmark for validating the docking protocol and for the comparative evaluation of the predicted binding affinity and interaction profiles of the newly identified candidate peptides. At the outset, MODELLER was employed to reconstruct the missing residues in the protein structure [46]. The structure was subsequently protonated: ionization states of titratable residues were predicted using PROPKA, and hydrogen atoms absent from the crystallographic model were added [44].

### 3.3. Peptide-ERK2 Docking Protocol

Binding poses of the chosen DRAMP-derived peptides within the ERK2 DRS were predicted using HADDOCK2.4 [47]. HADDOCK2.4 was selected for its information-driven docking approach, which enables the incorporation of experimentally established binding-site information through ambiguous interaction restraints (AIRs) while accounting for interfacial flexibility during structural refinement [47,48]. Given the availability of the crystallographically characterized ERK2-KIM-MAP3 complex, this approach was considered suitable for investigating peptide interactions within the predefined ERK2 DRS. This platform applies a data-driven docking strategy based on AIRs (ambiguous interaction restraints) inferred from anticipated interfacial residues. For docking estimations, critical ERK2 DRS residues, including THR116, LYS112, TYR129, TYR126, GLN313, THR157, ASP319, and TYR314, were specified as active interaction sites. On the peptide side, every residue was designated active and allowed full flexibility during refinement. It is worth mentioning that the HADDOCK protocol was designed to investigate potential peptide-binding modes within the predefined ERK2 DRS, rather than to establish preferential binding to this region over other potential sites on the ERK2 surface.

### 3.4. MD Simulations

Peptides exhibiting HADDOCK scores below −200.0 were selected for subsequent MDS using AMBER20 (version 2020) software [49]. This empirically defined threshold was employed as a practical criterion for prioritizing peptides with more favorable docking scores for further computational evaluation, rather than as a validated cutoff for distinguishing ERK2 DRS binders from non-binders. The employed MDS parameters were set as previously described [50,51,52,53]. In brief, ERK2 and the investigated DRAMP peptides were parameterized utilizing the AMBER force field 14SB [54]. The complexes were placed in truncated-octahedral TIP3P water boxes that extended at least 12 Å beyond the solute in all directions and contained roughly 13,000 water molecules [55]. Na^+^/Cl^−^ counterions were subsequently added in the numbers required to neutralize the net charge of each peptide-ERK2 complex. The prepared peptide-ERK2 complexes were minimized for 5000 cycles to remove unfavorable contacts. Following minimization, each peptide-ERK2 complex was gently heated to 310 K over a 50 ps interval and then equilibrated for 10 ns under the NPT ensemble. Production phases of 10, 50, and 200 ns were conducted for the peptide-ERK2 complexes, with snapshots saved every 10 ps. The pmemd.cuda module within AMBER20 was employed for all MDS. MDS were performed using GPU-accelerated nodes (pmemd.cuda), while other calculations were carried out on CPU-based nodes within the CompChem hybrid HPC cluster. Molecular visualizations were generated using the PyMOL (version 2.5.0) software package [56,57].

### 3.5. Binding Energy Assessment

The molecular mechanics-generalized Born surface area (MM-GBSA) approach was employed to compute the binding energies (Δ*G*_binding_) of the investigated peptides bound to the ERK2 DRS [58]. The Δ*G*_binding_ was estimated based on the following equation:

∆*G*_binding_ = *G*_Complex_ − *G*_Peptide_ − *G*_Receptor_

where the individual energy components (*G*) were obtained according to:

*G* = *G*_GB_ + *G*_SA_ + *E*_vdw_ + *E*_ele_

The van der Waals, electrostatic, generalized Born solvation, and surface area energy terms are denoted as *E*_vdW_, *E*_ele_, *G*_GB_, and *G*_SA_, respectively. The entropic contribution (−TΔ*S*) was excluded due to the substantial computational cost associated with its estimation [59,60]. Consequently, the calculated MM-GBSA binding energies were interpreted as approximate energetic estimates rather than absolute binding energies, with emphasis on relative energetic trends.

### 3.6. Physicochemical Characteristics

The physicochemical properties were further examined to provide a preliminary assessment of the drug-like properties of the identified peptides [61]. Physicochemical properties, including aliphatic index, MW (molecular weight), theoretical isoelectric point, instability index, and GRAVY (grand average of hydropathy), were computed employing the ProtParam server [62,63,64,65,66]. Proteins with an isoelectric point greater than 7.0 are classified as alkaline (basic), while those with isoelectric point values lower than 7.0 are classified as acidic. The instability index was used as an indicator of protein stability, with values exceeding 40.0 indicating unstable proteins, whereas values below 40.0 indicate stable proteins [67]. Defined as the relative volume contributed by aliphatic residues, the aliphatic index is commonly used as a proxy for thermal stability [64]. The GRAVY score provides a quantitative measure of a peptide’s overall hydrophobicity or hydrophilicity. Positive GRAVY values stand for a predominance of hydrophobic residues, whereas negative values indicate a higher contribution from hydrophilic residues.

## 4. Conclusions

ERK2 plays a central role in regulating multiple biological processes, and its aberrant signaling is strongly implicated in carcinogenesis and progression. Accordingly, selective inhibition of ERK2 via its DRS represents a promising conceptual strategy, pending experimental validation as a cancer medication. Herein, *in silico* screening approaches were employed to identify potential peptides targeting the DRS of ERK2. An initial set of 163 peptides annotated with anticancer activity was retrieved from the DRAMP database. Following sequence-based filtering, 31 peptides comprising 5–15 amino acids were selected for molecular docking against the ERK2 DRS. According to the predicted docking scores, the top-ranked peptides were subjected to a 200 ns MDS, along with their MM-GBSA calculations. Upon MM-GBSA//200 ns MDS, DRAMP03987, DRAMP03983, DRAMP03985, and KIM-MAP3 displayed promising binding affinities against the ERK2 DRS, with Δ*G*_binding_ values of −71.2, −65.1, −63.1, and −51.5 kcal/mol, respectively. Furthermore, structural and energetic analyses disclosed that the identified peptides maintained stable interaction profiles within the ERK2 DRS throughout 200 ns MDS. In addition, the predicted physicochemical profiles of the identified peptides further highlighted their preliminary physicochemical characteristics. The present findings are based on computational predictions and do not establish direct binding to the ERK2 DRS, functional inhibition, or anticancer efficacy. Therefore, further experimental investigations involving biophysical binding assays, substrate-competition studies, kinase activity measurements, and cellular assays are warranted to validate the predicted peptide-ERK2 interactions and assess their functional and biological significance.

## Figures and Tables

**Table 1:** Computed docking scores and intermolecular H-bonds of the three most potent DRAMP peptides and KIM-MAP3 towards ERK2 DRS.

Peptide ID	Amino Acid Sequence	Docking Score (kcal/mol)	Intermolecular H-Bonds (Å)
KIM-MAP3	RRLQKGNLPVR	–322.1	ASP319-ARG65 (1.5 Å), LYS112-ARG74 (1.6, 1.8 Å), TYR126-LEU66 (2.7 Å), THR116-ARG74 (2.5 Å), TYR314-GLN67 (2.1 Å), THR157-LEU71 (1.9 Å), TYR129-ARG64 (2.3 Å), GLN313-LYS68 (1.7 Å), TYR314-ARG64 (2.1, 2.5 Å)
DRAMP03983	KWKLFKKIPKFLHLA	−318.6	GLU107-LYS10 (1.5 Å), THR157-LYS10 (1.8 Å), THR157-ILE8 (1.8 Å), TYR129-LYS3 (2.2 Å), ASP319-LYS3 (1.5 Å), TYR315-LYS1 (1.6 Å), GLU312-LYS1 (1.7, 1.6 Å), TYR314-TRP2 (2.1 Å), TYR314-LEU4 (1.8 Å)
DRAMP03987	KWKLFKKIPKFLH	−292.3	ASP122-LYS6 (1.5, 2.2 Å), TYR314-LYS6 (1.7 Å), THR157-ILE8 (1.9 Å), ASP316-LYS1 (1.5 Å), ASP319-LYS1 (1.5 Å)
DRAMP03985	KWKLFKKIPKFLHL	−273.9	LYS112-LEU14 (2.5 Å), LYS112-LEU12 (1.6 Å), SER120-LYS6 (1.7 Å), SER120-HIS123 (1.7 Å), TYR126-LYS3 (1.7 Å), TYR314-LYS1 (2.4 Å), TYR314-TRP2 (1.7 Å), TYR315-LYS1 (2.5 Å), ASP316-LYS1 (2.0 Å)

**Table 2:** Predicted physicochemical features of DRAMP03987, DRAMP03983, DRAMP03985, and KIM-MAP3.

Peptides ID	MW	Theoretical Isoelectric Points	Estimated Half-Life	Instability Index	Aliphatic Index	GRAVY
KIM-MAP3	1336.61	12.30	1 h (mammalian reticulocytes, *in vitro*), 2 min (yeast, *in vivo*), 2 min (*Escherichia coli*, *in vivo*)	110.38	97.27	−1.327
DRAMP03987	1713.19	10.60	1.3 h (mammalian reticulocytes, *in vitro*), 3 min (yeast, *in vivo*), 3 min (*Escherichia coli*, *in vivo*)	−17.61	90.00	−0.577
DRAMP03983	1897.43	10.60	1.3 h (mammalian reticulocytes, *in vitro*), 3 min (yeast, *in vivo*), 3 min (*Escherichia coli*, *in vivo*)	−13.93	110.67	−0.127
DRAMP03985	1826.35	10.60	1.3 h (mammalian reticulocytes, *in vitro*), 3 min (yeast, *in vivo*), 3 min (*Escherichia coli*, *in vivo*)	−15.64	111.43	−0.264

## Data Availability

The original contributions presented in this study are included in the article/Supplementary Materials. Further inquiries can be directed to the corresponding authors.

## Supplementary Materials

The following supporting information can be downloaded at: https://www.mdpi.com/article/10.3390/ijms27219532/s1.
