# Supplementary material for: Targeting the ERK2 D-Recruitment Site with Anticancer Peptides from the DRAMP Database: An Integrated In Silico Study
Source: Int J Mol Sci. 2026 Oct 25;27(21):9532. doi: 10.3390/ijms27219532 (PMC13763456; doi:10.3390/ijms27219532)
Supplement: Supplementary file 1 [file ijms-27-09532-s001.zip › ijms-4628154-supplementary.pdf]

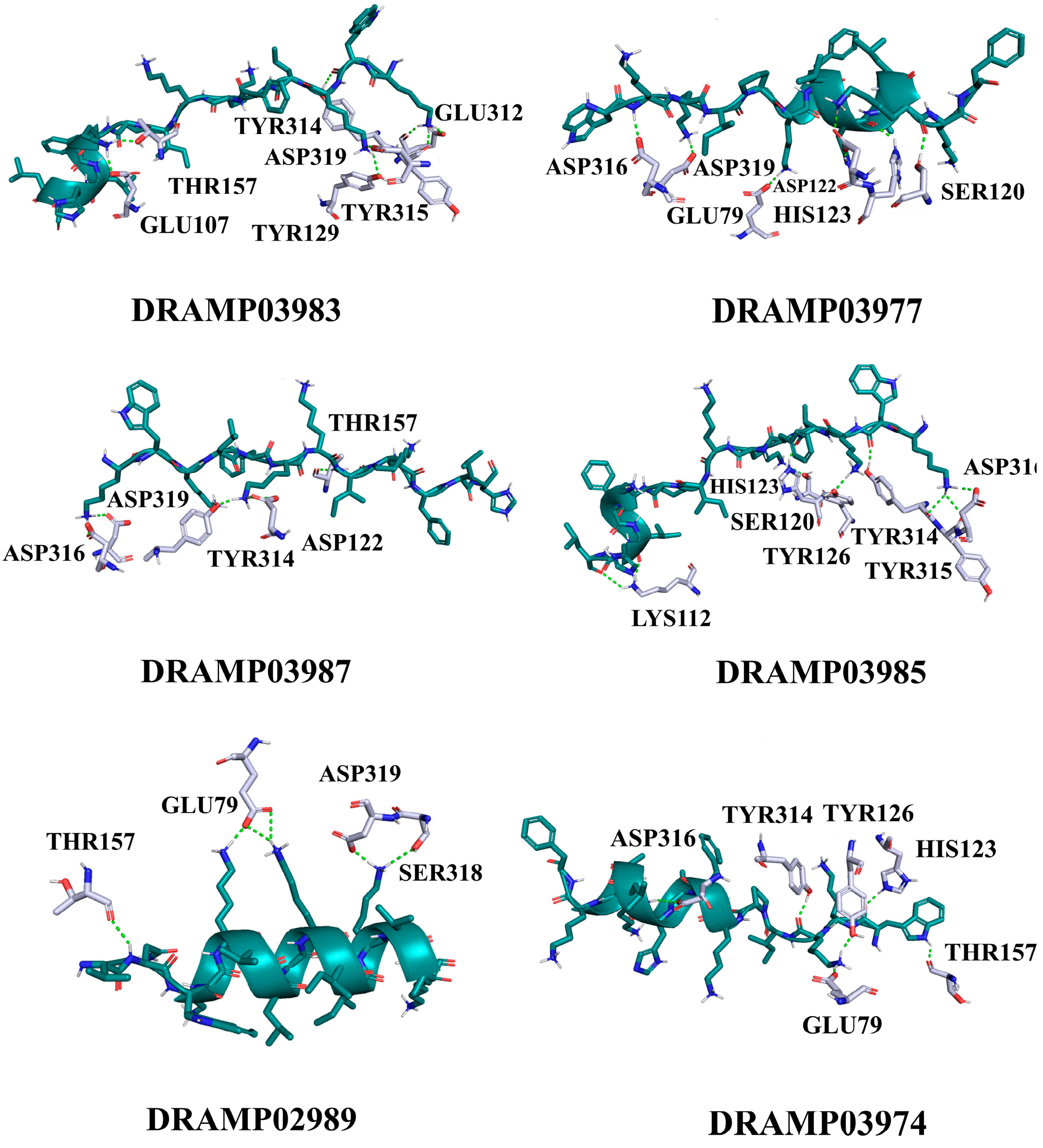

Figure S1. 3D molecular interaction patterns of the predicted binding modes for the investigated 31 DRAMP peptides within ERK2 DRS**.**

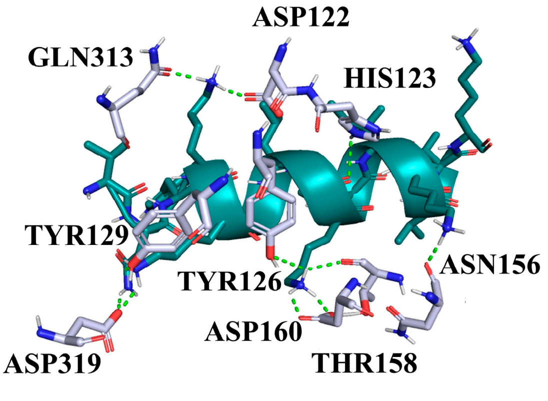

DRAMP02991

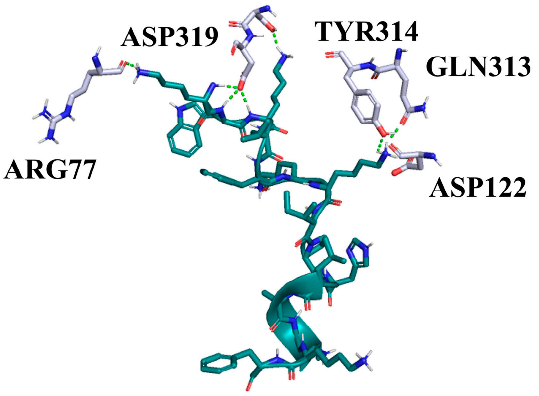

DRAMP03982

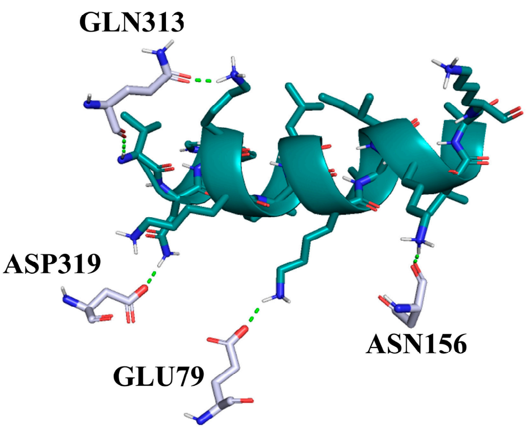

DRAMP02990

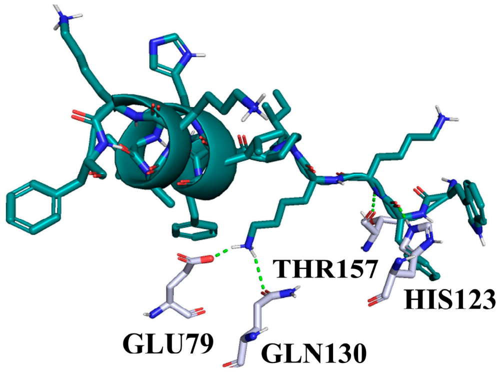

DRAMP03973

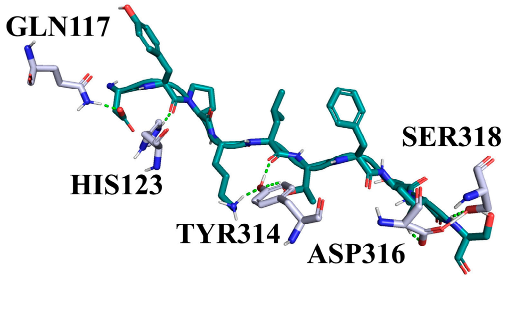

DRAMP00301

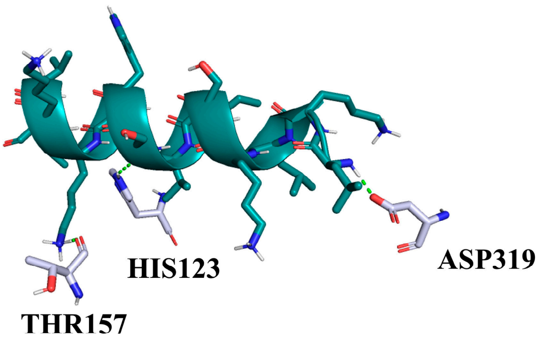

DRAMP03024

Figure S1. *Continued.*

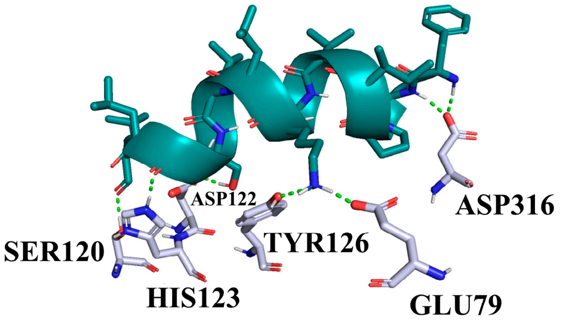

DRAMP18658

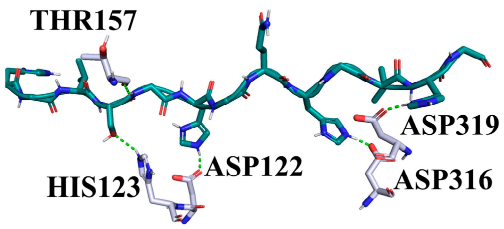

DRAMP03064

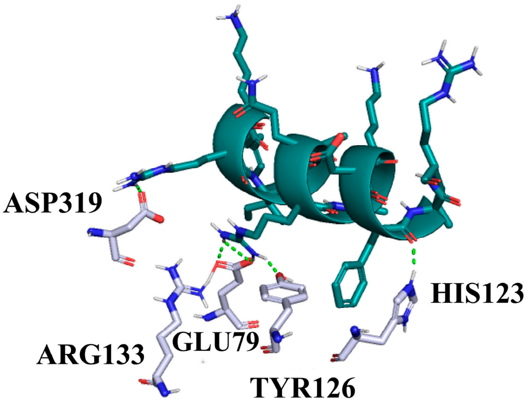

DRAMP03575

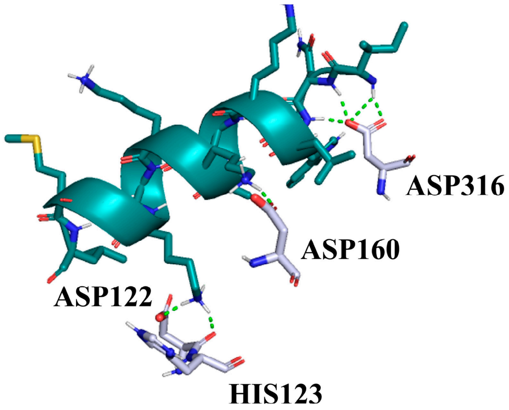

DRAMP29088

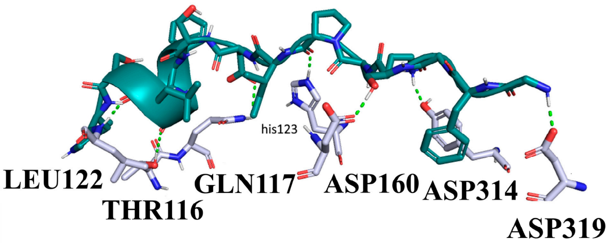

DRAMP18366

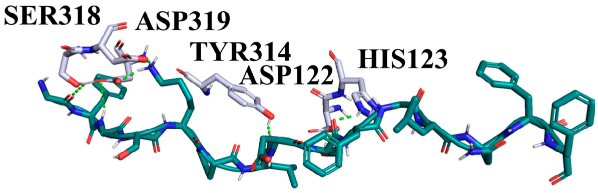

DRAMP18367

Figure S1. *Continued.*

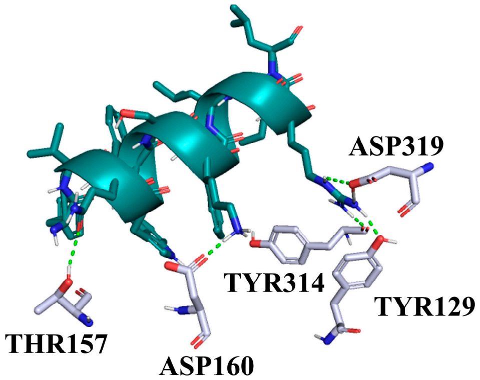

DRAMP01746

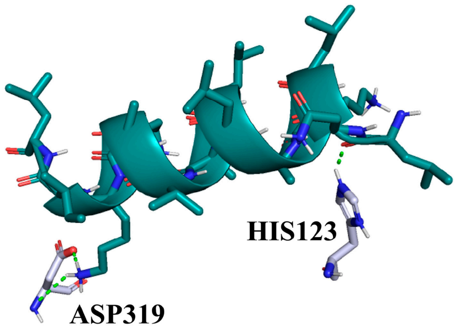

DRAMP29104

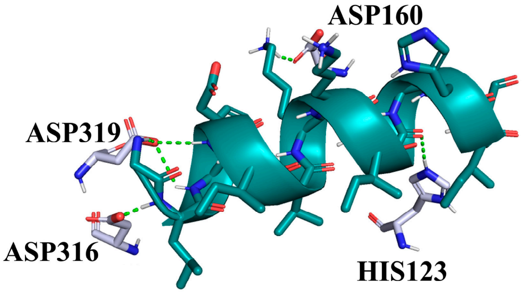

DRAMP01615

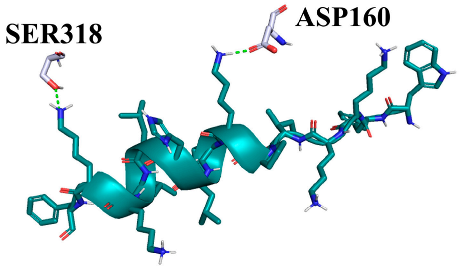

DRAMP03976

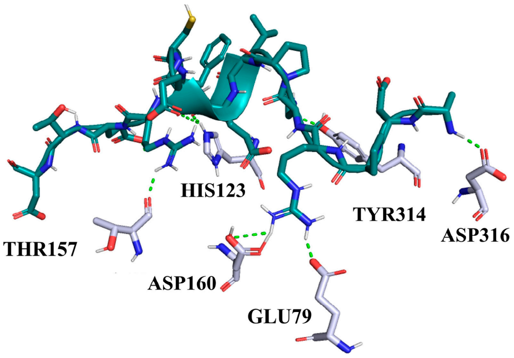

DRAMP18132

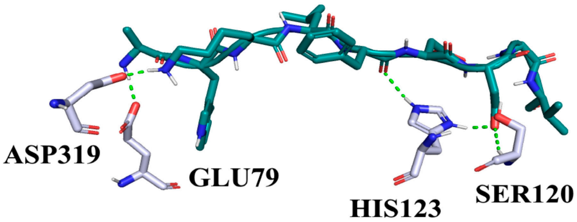

DRAMP01064

Figure S1. *Continued.*

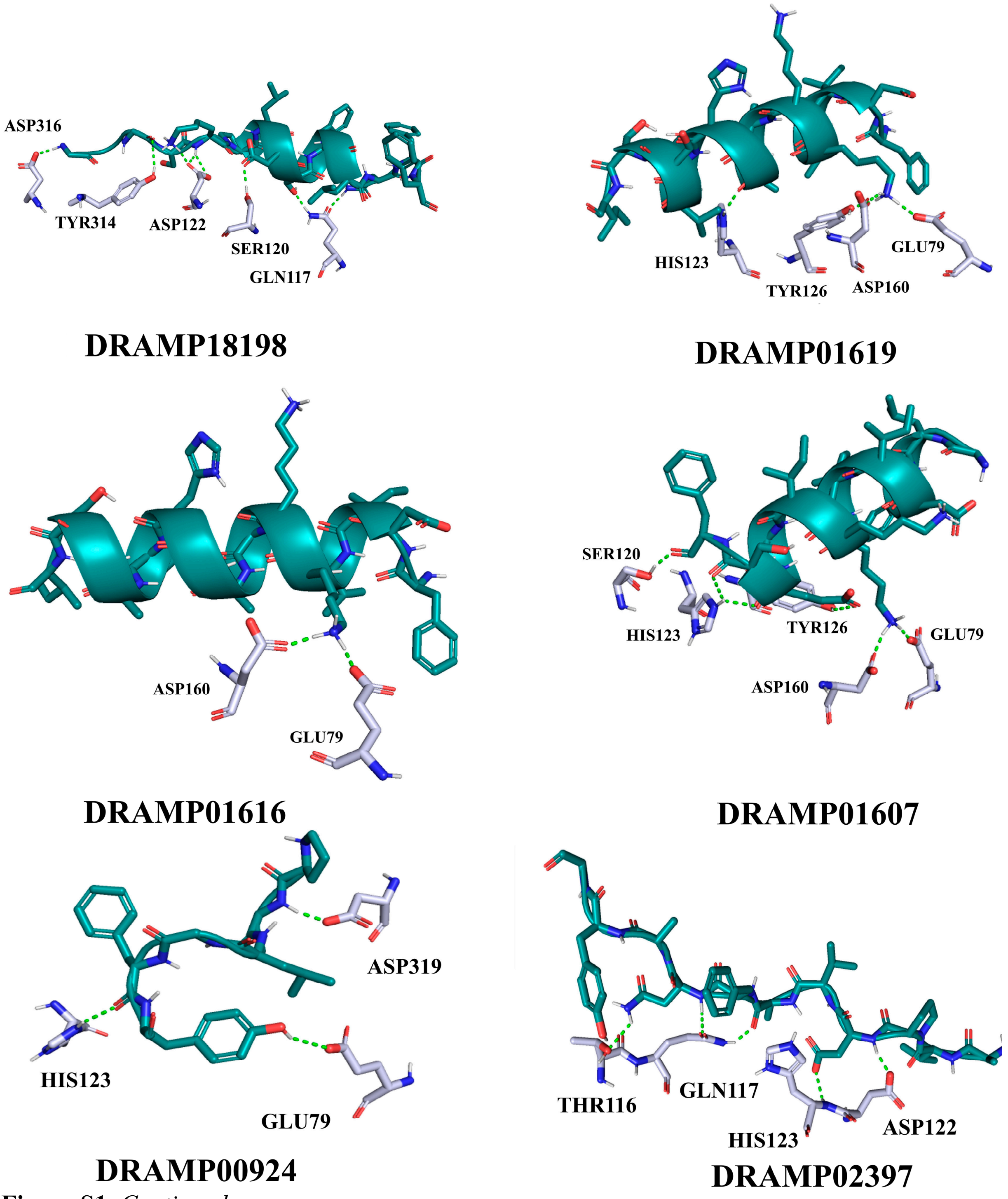

Figure S1. *Continued.*

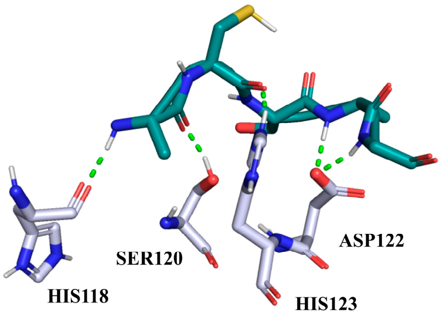

DRAMP02396

Figure S1. *Continued*.

Table S1. Overview of primary sequences and peptide lengths of the selected DRAMP peptides with anticancer potential.

Peptide Name/Code	Sequence Length	Amino Acid Sequence	Peptide Name/Code	Sequence Length	Amino Acid Sequence
**KIM-MAP3**	11	RRLQKGNLPVR	DRAMP03977	14	WKKIPKFLHLLKKF
DRAMP02396	5	ACSAG	DRAMP03985	14	KWKLFKKIPKFLHL
DRAMP00924	6	PGLGFY	DRAMP29088	14	INWLKIAKKVKGML
DRAMP01064	9	AWKLFDDGV	DRAMP18367	15	GFGSKPLDSFGLNFF
DRAMP00301	10	DYPKLTFTTS	DRAMP18366	15	GFGSKPIDSFGLSWL
DRAMP02397	11	AVPDVAFNAYG	DRAMP01616	15	FDIVKKIAGHIAGSI
DRAMP01607	13	GLFDIIKKIAESF	DRAMP01619	15	FDIVKKIAGHIVSSI
DRAMP01746	13	FVQWFSKFLGRIL	DRAMP02989	15	VNWKKVLGKIIKVAK
DRAMP03575	13	FKRIVQRIKDFLR	DRAMP02990	15	VNWKKILGKIIKVAK
DRAMP18658	13	FLPIVAKLLSGLL	DRAMP02991	15	VNWKKILGKIIKVVK
DRAMP03987	13	KWKLFKKIPKFLH	DRAMP18198	15	GFGSKPLDSFGLNFF
DRAMP03064	13	HGVSGHGQHGVHG	DRAMP03973	15	WFKKIPKFLHLAKKF
DRAMP03024	14	LKLKSIVSWAKKVL	DRAMP03976	15	WFKKIPKFLHLLKKF
DRAMP01615	14	GLFDIVKKIAGHIA	DRAMP03982	15	KWKLFKKIPHLAKKF
DRAMP29104	14	LNLKALLAVAKKIL	DRAMP03983	15	KWKLFKKIPKFLHLA
DRAMP03974	14	WKKIPKFLHLAKKF	DRAMP18132	15	ADPRNPLEECFRETD

Table S2. Predicted docking scores of the investigated 31 DRAMP peptides and KIM-MAP3 against ERK2 DRS.

Peptide ID/Name	Docking score (kcal/mol)	Peptide ID/Name	Docking score (kcal/mol)
KIM-MAP3	−322.1	DRAMP29088	−209.6
DRAMP03983	−318.6	DRAMP18366	−209.6
DRAMP03987	−292.3	DRAMP18367	−208.4
DRAMP03977	−279.8	DRAMP01746	−207.9
DRAMP03985	−273.9	DRAMP29104	−206.8
DRAMP02989	−271.0	DRAMP01615	−206.3
DRAMP03974	−267.1	DRAMP03976	−193.9
DRAMP02991	−252.6	DRAMP18132	−188.1
DRAMP03982	−251.5	DRAMP01064	−179.9
DRAMP02990	−240.7	DRAMP18198	−165.6
DRAMP03973	−234.9	DRAMP01619	−160.9
DRAMP00301	−233.0	DRAMP01616	−160.4
DRAMP03024	−224.7	DRAMP01607	−157.1
DRAMP18658	−224.5	DRAMP00924	−155.9
DRAMP03064	−215.2	DRAMP02397	−142.5
DRAMP03575	−210.1	DRAMP02396	−85.6

Table S3. Estimated MM-GBSA binding energies (in kcal/mol) over 10 ns MDS for the investigated 21 DRAMP peptides and KIM-MAP3 within the ERK2 DRS.

No.	Peptide ID/Name	MM-GBSA Binding Energies (kcal/mol)
	**KIM-MAP3**	**−46.9**
1	DRAMP03987	−52.6
2	DRAMP03983	−57.2
3	DRAMP03985	−53.6
4	DRAMP02991	−53.5
5	DRAMP02990	−47.8
6	DRAMP01746	−46.8
7	DRAMP18366	−45.2
8	DRAMP03973	−41.5
9	DRAMP03977	−40.0
10	DRAMP18367	−39.2
11	DRAMP03982	−38.3
12	DRAMP03974	−33.6
13	DRAMP03575	−32.3
14	DRAMP01615	−29.3
15	DRAMP03024	−28.9
17	DRAMP29088	−28.1
18	DRAMP18658	−24.9
19	DRAMP02989	−19.8
20	DRAMP00301	−19.8
21	DRAMP03064	−19.0

Table S4. Computed MM-GBSA binding energies from two independent 50 ns MDS for the most potent DRAMP peptides and KIM-MAP3 bound to the ERK2 DRS.

Peptide ID	MM-GBSA binding energy (kcal/mol)	
	**1st**	**2nd**
**KIM-MAP3**	–44.8±9.3	–48.0±11.2
DRAMP03987	–65.5±9.6	–61.5±7.5
DRAMP03983	–58.7±8.0	–59.9±6.8
DRAMP03985	–57.2±6.5	–55.7±9.4
